# Supplementary material for: Dynamics and diversity in adolescents’ experienced barriers and facilitators for physical activity maintenance
Source: PLoS One. 2025 Sep 23;20(9):e0333120. doi: 10.1371/journal.pone.0333120 (PMC12456830; doi:10.1371/journal.pone.0333120)

S1 Figure. Materials used during interviews to sensitize adolescents about their active lifestyle.

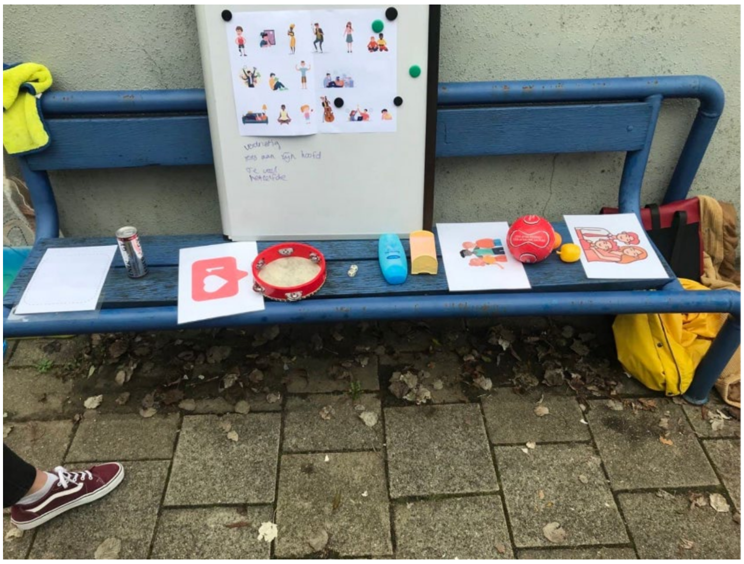

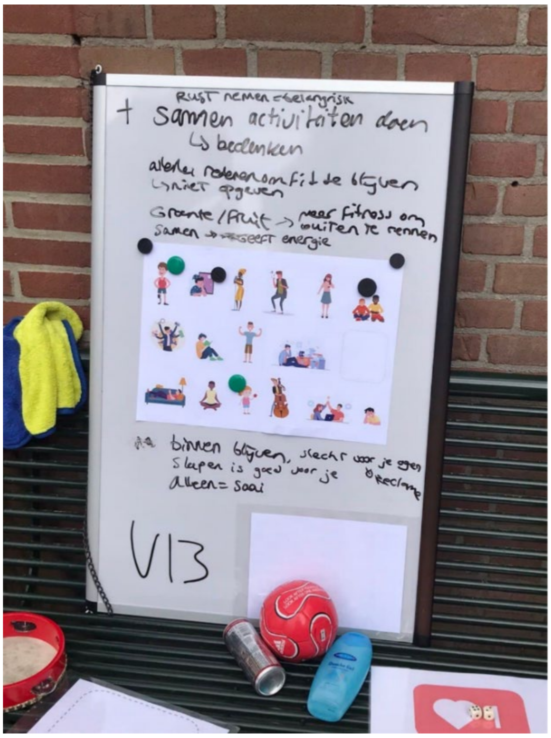

Supplement: S1 Fig — (PDF) [file pone.0333120.s001.pdf]
