# Supplementary material for: Dynamics and diversity in adolescents’ experienced barriers and facilitators for physical activity maintenance
Source: PLoS One. 2025 Sep 23;20(9):e0333120. doi: 10.1371/journal.pone.0333120 (PMC12456830; doi:10.1371/journal.pone.0333120)
Supplement: S2 Table — (PDF) [file pone.0333120.s002.pdf]

S2 Table A. Final list of facilitators used during Q-sorting.

#	Facilitator	Theme
1	Able to be myself	**Intrapersonal**
2	Able to do something that others can't	
3	For my future	
4	Feeling strong	
5	Looking good	
6	Clearing my mind	
7	Feeling good in my body	
8	Competing against others	
9	Learning new things	
10	Releasing my energy	
11	Having good conditioning	
12	Reaching my goals	
13	Making my own choices	
14	Developing myself	
15	Gaining self-confidence	
16	Feeling energized	
17	It's a habit	
18	For my health	
19	Support from family/parents	**Interpersonal**
20	Avoiding punishment	
21	Being like a role model	
22	Encouragement from others	
23	Being part of a team	
24	Meeting (new) friends	
25	Doing things with others	
26	Having fun	**Activity nature**
27	Physicality of sports	
28	Challenge	
29	A lot of variation	
30	Good guidance	
31	Easily combined with other activities	**Life factors**
32	A balanced life	
33	Wanting to maintain something	
34	Good weather	**(Environmental) resources**

35	In my neighborhood	
36	Environment in which I feel safe	

S2 Table B. Final list of barriers used during Q-sorting.

#	Barrier	Theme
1	Too much on my mind	**Intrapersonal**
2	Too many other distractions	
3	Not feeling well	
4	Feeling lazy	
5	Low energy	
6	Not wanting to go too hard	
7	No motivation (anymore)	
8	Not as expected	
9	Had a bad experience	
10	It doesn't fit me	
11	Not enough freedom	
12	Having to choose	
13	Got out of the rhythm	
14	Not being good enough	
15	Forgot my (long-term) goals	
16	Being judged by others	**Interpersonal**
17	No support from parents/family	
18	Perceiving pressure from others	
19	Being alone	
20	No fun	**Activity nature**
21	Not the right level	
22	Not seeing progress	
23	Lack of time	**Life factors**
24	Other responsibilities	
25	Bad weather	**(Environmental) resources**
26	Distance	
27	Preparation is annoying	
28	Too expensive	
29	Not enough options	
30	Not the right material	
31	Unsafe environment	
