# Supplementary material for: Dynamics and diversity in adolescents’ experienced barriers and facilitators for physical activity maintenance
Source: PLoS One. 2025 Sep 23;20(9):e0333120. doi: 10.1371/journal.pone.0333120 (PMC12456830; doi:10.1371/journal.pone.0333120)
Supplement: S3 Table — (PDF) [file pone.0333120.s003.pdf]

S3 Table. The different types of physical activities that adolescents had maintained.

Activity	Participants
Football	5
Gymnastics	4
Dancing	4
Martial arts	3
Tennis	3
Biking	2
Field hockey	2
Basketball	2
Fitness	2
Sport climbing	1
Skateboarding	1
Playing outdoors	1
