# Supplementary material for: Dynamics and diversity in adolescents’ experienced barriers and facilitators for physical activity maintenance
Source: PLoS One. 2025 Sep 23;20(9):e0333120. doi: 10.1371/journal.pone.0333120 (PMC12456830; doi:10.1371/journal.pone.0333120)
Supplement: S4 Table — (PDF) [file pone.0333120.s004.pdf]

S4 Table A. Weighted average z-scores per facilitator factor.

	Factor				
**Facilitator**	**A**	**B**	**C**	**D**	**E**
Learning new things	0	0	1	0	0
Doing things with others	2	2	0	0	0
Feeling strong	0	0	0	2	0
Feeling energized	1	0	0	0	2
A balanced life	0	0	2	0	0
Being part of a team	1	1	0	0	0
Meeting (new) friends	0	1	0	0	0
Challenge	0	0	2	0	0
Able to be myself	0	4	0	4	0
Having fun	3	3	1	0	3
Support from family/parents	0	2	1	2	1
For my health	2	0	3	0	0
Reaching my goals	0	0	4	2	0
Good guidance	0	1	0	1	0
Wanting to maintain something	0	0	2	1	0
Having good conditioning	1	0	3	0	1
Clearing my mind	4	0	0	1	0
In my neighborhood	0	0	0	0	1
For my future	0	0	0	0	2
Competing against others	0	0	0	0	0
Releasing my energy	3	0	0	0	0
Feeling good in my body	1	2	0	1	0
It's a habit	0	0	0	0	1
Gaining self-confidence	0	0	0	3	0
Developing myself	0	0	1	3	4
Making my own choices	0	1	0	0	3
Physicality of sports	2	0	0	0	2
Environment in which I feel safe	0	3	0	0	0
Able to do something that others can't	0	0	0	0	0
Looking good	0	0	0	0	0
Avoiding punishment	0	0	0	0	0
Being like a role model	0	0	0	0	0
Encouragement from others	0	0	0	0	0
A lot of variation	0	0	0	0	0
Easily combined with other activities	0	0	0	0	0
Good weather	0	0	0	0	0

Note: A = ‘’, B = ‘’, C = ‘’, D = ‘’, E = ‘’ and F = ‘’. “0” indicates that the adolescents in that factor on (weighted) average found that *facilitator not important*; “4” indicates adolescents in that factor on (weighted) average found that *facilitator most important*. 0 in all columns means that this facilitator was not significantly associated with any factor.

S4 Table B. Weighted average z-scores per barrier factor.

	Factor			
**Barrier**	**A**	**B**	**C**	**D**
Not enough options	0	0	1	0
Other responsibilities	1	1	4	3
Unsafe environment	0	4	0	0
Bad weather	0	0	0	2
Distance	0	0	0	4
Got out of the rhythm	1	0	3	1
Not feeling well	3	0	0	3
Lack of time	2	3	2	0
Perceiving pressure from others	0	0	0	1
Low energy	3	0	0	0
No fun	1	2	0	0
Not the right level	0	0	0	2
Being judged by others	0	0	0	2
It doesn't fit me	0	3	0	0
Not enough freedom	0	1	0	0
No motivation (anymore)	4	0	1	0
Had a bad experience	0	1	1	1
Too expensive	0	2	0	0
Not wanting to go too hard	0	0	1	0
Having to choose	0	0	2	0
Feeling lazy	2	0	3	0
Too much on my mind	2	2	0	1
Too many other distractions	1	0	2	0
No support from parents/family	0	1	0	0
Not being good enough	0	0	0	0
Forgot my (long-term) goals	0	0	0	0
Not as expected	0	0	0	0
Preparation is annoying	0	0	0	0
Not the right material	0	0	0	0
Being alone	0	0	0	0
Not seeing progress	0	0	0	0

Note: A = ‘’, B = ‘’, C = ‘’ and D = ‘’. “0” indicates that the adolescents in that factor on (weighted) average found that *barrier not important*; “4” indicates adolescents in that factor on (weighted) average found that *barrier most important*. 0 in all columns means that this barrier was not significantly associated with any factor.
