# Supplementary material for: Dynamics and diversity in adolescents’ experienced barriers and facilitators for physical activity maintenance
Source: PLoS One. 2025 Sep 23;20(9):e0333120. doi: 10.1371/journal.pone.0333120 (PMC12456830; doi:10.1371/journal.pone.0333120)
Supplement: Attachment — Submitted filename: comments to authors.pdf [file pone.0333120.s005.pdf]

This is an interesting piece of work, using interesting methods of engaging with young people. The exploration of PA maintenance is important. Below are some more specific comments I think need to be addressed to strengthen the manuscript. I have selected major revision purely because some points may need some time and consideration to be addressed.

Abstract

- It would be nice to state what the five distinct facilitator clusters and four barrier clusters were e.g. titles

Introduction

- The authors provide the statistic of 81% adolescents not engaging with PA – it would be interesting to note how that split differs by gender
- "when an intervention is finalized" – what do the authors mean by this? I think the sentence needs to be rephrased for clarity
- The introduction could do with some restructuring, starting of broader and then going into a more specific lens. At the moment it is a bit difficult to follow the 'flow' of it. In the beginning it goes from saying interventions aren't successful and then talks about PA more generally... there is a huge jump and gap there that could do with more consideration. The introduction continues to then jump back and forth, and discuss some points that do not seem relevant (and may be better suited to the discussion). The description and lead to the need to investigate PA maintenance is important, and justifies the project so some more clarity and restructuring for flow around this could strengthen this section of the paper.
- At the end of the introduction there is a summary of the methods chosen which I would argue is not needed – simply lead to the aim of the paper and then talk about the methods in the methods section only

Methods

- Not a deal breaker but the process of obtaining informed consent could be under a separate subheading
- The recruitment process is currently also under the ethics subheading – this needs to be separated in my opinion, with further detail added about the recruitment process (and this could be reflected on in the discussion...). I also think numbers approached, numbers agreeing to take part should be included here. Description on how participants were approached, the timing of everything, the where etc need to be included... were they sent home with a

letter, and then did they contact the research team who would then show up at school for the interview?

- Gender does not seem to be reported even though the authors stated this information was collected. Only the age is reported. Please report the gender of the participants
- It would be good if the authors collected data on / could reflect on whether the adolescents they interviewed were active currently
- I think it is important, for the discussion around transferring/generalising findings, if there is some section/ consideration of the context (e.g. as mentioned above PA engagement of the adolescents)... and perhaps some consideration of the biases/background of the researchers and some reflection of /acknowledgement of the impact of this on the results generated
- Some justification of the Q method and what this method/approach offers that another e.g. a thematic analysis, grounded theory etc does not offer... i.e. why a Q methodology?

Results

- I do like the consideration of factors outside of individuals – it is a shame these are supplementary I think there should be more consideration of them and discussion of the implications of this within the paper itself
- I like the visual way results are presented in the figures – something different! Note to the editor to make sure these are printed/accessible clearly
- Once question I have and it may be that I have missed it – the results talk about changes but the barriers and facilitators presented seem to be a snapshot? Can the authors clarify exactly how they have come to the understanding of the changes and PA maintenance? I can see from figure 2 there is consideration there... is this totally separate to or linked with the barriers and facilitators presented in figures 3 and 4?

Discussion

- I do think there is something missing here.. in how can changes in perceptions over time about PA be discussed without the context of / understanding about previous vs current PA levels? Or the impact on these...
- *"When investigating adolescents' prior experience of PA maintenance, we observed a notable shift."* What is this shift? Be specific...
- This paragraph starting: *"Using cluster analysis on the card sorting task resulted in five facilitator and four*

- *barrier clusters for PA maintenance. These clusters illustrate that adolescents' barriers and facilitators to PA maintenance are personally specific, with different groups experiencing…"* is quite short and missing depth. Either needs to be incorporated elsewhere or expended
- some of the paragraphs just list the results and state other papers that agree with this… are there any other papers out there that disagree? Could the implications / so what of these be discussed further…
- There needs to be a defined and separate limitations section within the discussion, specifically there could be some discussion of how some of the artefacts used could be leading – what was chosen to represent the 'who is fitter' Q that was given etc. and/or the consideration of different ages, being at a different life stage and how this may impact perceptions. What I mean is how can you compare the change over time from 10 to 13 years old versus 12 to 17 years old… this is a different transition, could details/understanding/voices be lost? The authors own reflections of the potential limitations and what the study offers should therefore be considered and included.
