# Supplementary material for: Dynamics and diversity in adolescents’ experienced barriers and facilitators for physical activity maintenance
Source: PLoS One. 2025 Sep 23;20(9):e0333120. doi: 10.1371/journal.pone.0333120 (PMC12456830; doi:10.1371/journal.pone.0333120)
Supplement: Attachment — Submitted filename: PlosOne response letter_TAH.docx [file pone.0333120.s007.docx]

Response letter PlosOne submission

**Title:**

**Dynamics and Diversity in Adolescents’ Experienced Barriers and Facilitators for Physical Activity Maintenance**

**All comments by reviewer:**

This is an interesting piece of work, using interesting methods of engaging with young

people. The exploration of PA maintenance is important. Below are some more specific

comments I think need to be addressed to strengthen the manuscript. I have selected

major revision purely because some points may need some time and consideration to

be addressed.

*Thank you for your comments and your time. We believe that the revisions have made the paper significantly better.*

**Abstract**

It would be nice to state what the five distinct facilitator cluster and four barrier

clusters were e.g. titles

The titles of the clusters are now mentioned in the abstract. We did also find a reporting mistake. Since the analysis we did a factor analysis (as opposed to a cluster analysis) the output should be referred to as factors in the manuscript. We have altered it throughout the writing and use factors and profiles interchangeably.

**Introduction**

The authors provide the statistic of 81% adolescents not engaging with PA – it

would be interesting to note how that split differs by gender

These differences are now stated.

“when an intervention is finalized” – what do the authors mean by this? I think

the sentence needs to be rephrased for clarity

We clarified this sentence to clearly state that it is about the longer-term follow-up adherence to PA that persist after the intervention period has ended.

The introduction could do with some restructuring, starting of broader and then

going into a more specific lens. At the moment it is a bit difficult to follow the

‘flow’ of it. In the beginning it goes from saying interventions aren’t successful

and then talks about PA more generally… there is a huge jump and gap there that

could do with more consideration. The introduction continues to then jump back

and forth, and discuss some points that do not seem relevant (and may be better

suited to the discussion). The description and lead to the need to investigate PA

maintenance is important, and justifies the project so some more clarity and

restructuring for flow around this could strengthen this section of the paper.

The introduction was restructured to follow a more logical flow from broad to specific. We shortened the text for conciseness and reordered the topics. In the previous version, changes in barriers and facilitators were discussed before their interaction. In the revised version, we first address the interaction between barriers and facilitators, then describe how they change over time. To maintain consistency throughout the text, the results and discussion sections were also reordered to reflect this structure.

At the end of the introduction there is a summary of the methods chosen which I

would argue is not needed – simply lead to the aim of the paper and then talk

about the methods in the methods section only

The summary of the methods has been removed from the introduction.

**Methods**

Not a deal breaker but the process of obtaining informed consent could be under

a separate subheading

We describe this process now under the ethics subheading

The recruitment process is currently also under the ethics subheading – this

needs to be separated in my opinion, with further detail added about the

recruitment process (and this could be reflected on in the discussion…). I also

think numbers approached, numbers agreeing to take part should be included

here. Description on how participants were approached, the timing of

everything, the where etc need to be included… were they sent home with a

letter, and then did they contact the research team who would then show up at

school for the interview?

The recruitment process is now presented under a separate subheading and described in greater detail. We do not have data on how many students were approached or declined participation. Information for parents and guardians was sent via the schools’ digital messaging systems, so we don’t have the exact number reached.

Gender does not seem to be reported even though the authors stated this

information was collected. Only the age is reported. Please report the gender of

the participants

Gender and age of participants is now reported more clearly.

It would be good if the authors collected data on / could reflect on whether the

adolescents they interviewed were active currently

For the phase 2 card sorting task, we selected students who reported being physically active for at least 2 years in one activity, although we did not measure their exact activity levels. For the interviews of phase 1 we did not have this criteria because it was to get a more general sense of barriers and facilitators. This is now also described more clearly in the methods section.

I think it is important, for the discussion around transferring/generalising

findings, if there is some section/ consideration of the context (e.g. as mentioned

above PA engagement of the adolescents)… and perhaps some consideration of

the biases/background of the researchers and some reflection of

/acknowledgement of the impact of this on the results generated

Thank you for this important comment. In the discussion and limitations section, we have added further reflections on the generalizability of our findings and the influence of the local context. We also elaborate on our own biases and assumptions and how these may have affected the study’s design, results, and interpretation.

Some justification of the Q method and what this method/approach offers that

another e.g. a thematic analysis, grounded theory etc does not offer… i.e. why a

Q methodology?

We have expanded our justification for using Q methodology in this study and explained in greater detail how it fits with our research aims.

**Results**

do like the consideration of factors outside of individuals – it is a shame these

are supplementary I think there should be more consideration of them and

discussion of the implications of this within the paper itself

We are not entirely sure what is meant by “supplementary” in this context (e.g., whether it refers to the supplementary materials?). In any case, one relevant consideration is that most barriers and facilitators are related to intrapersonal barriers/facilitators. That said, we have rephrased the discussion section to better reflect the role of both personal factors and those outside the self, as we consider this an important aspect.

I like the visual way results are presented in the figures – something different!

Note to the editor to make sure these are printed/accessible clearly

Thank you for this positive comment on our experiment! We made some adjustment to the layout and look of the figures to ensure easier clear printing.

Once question I have and it may be that I have missed it – the results talk about

changes but the barriers and facilitators presented seem to be a snapshot? Can

the authors clarify exactly how they have come to the understanding of the

changes and PA maintenance? I can see from figure 2 there is consideration

there… is this totally separate to or linked with the barriers and facilitators

presented in figures 3 and 4?

In Figure 2 (now renumbered to Figure 4 due to text reordering; see previous comment), we focus on changes over time, based on adolescents’ retrospective reflections. These represent perceived, rather than measured, changes over time. We have refined the language throughout the paper to make this distinction clearer and improve transparency.

Figures 3 and 4 (now Figures 2 and 3) present snapshots of the present. We attempted to create factors based on participants’ retrospective configurations, but the sample size and the way retrospection was applied did not yield coherent or informative results. For example, when reflecting on past experiences, some participants removed certain items from their rankings, resulting in substantial missing data that prevented a robust factor analysis.

**Discussion**

I do think there is something missing here.. in how can changes in perceptions

over time about PA be discussed without the context of / understanding about

previous vs current PA levels? Or the impact on these…

Thank you for highlighting this important point. We have added more reflections in the discussion on how these perceived changes relate to PA maintenance. Our main aim was to illustrate that even when adolescents maintain a physical activity, their barriers and facilitators can, and perhaps must, change over time to support continued engagement. This concept is now also introduced more clearly in the introduction.

In our phase 2 sample, our inclusion criteria was that they had done an activity for at least 2 years regularly and unfortunately we did not record whether they were more or less active in the past compared to the present. We acknowledge that this is an assumption may not fully reflect the reality of their PA levels. Our focus in this study was on adolescents’ experiences and perceptions of maintaining PA, rather than on activity intensity itself. We fully agree that to obtain a more complete picture of adolescent PA, our approach should be complemented with additional data, such as self-reported or objectively measured activity levels, a point now stated more clearly in the limitations section.

Nonetheless, we believe this study still offers valuable insights into adolescents’ experiences of PA maintenance.

“When investigating adolescents’ prior experience of PA maintenance, we

observed a notable shift.” What is this shift? Be specific…

We have clarified in the discussion that our focus is specifically on participants’ retrospective perceptions of barriers and facilitators and what exactly these changes include.

This paragraph starting: “Using factor analysis on the card sorting task resulted

in five facilitator and four barrier clusters for PA maintenance. These clusters illustrate that adolescents' barriers and facilitators to PA maintenance are personally specific, with different

groups experiencing…” is quite short and missing depth. Either needs to be

incorporated elsewhere or expended

We have expanded this paragraph to move beyond simply repeating the results. It now offers additional depth by interpreting the factors through different lenses such as the immediate vs. future-oriented dimension for facilitators and internal vs. external barriers.

some of the paragraphs just list the results and state other papers that agree

with this… are there any other papers out there that disagree? Could the

implications / so what of these be discussed further…

We added more critical notes in the discussion to enhance the transparency of the work. We added some different perspectives (e.g. other components for maintenance that we don’t address or only indirectly) to make the discussion more balanced and less one-sided. The implications of the findings are now discussed in more detail rather than just mentioning sources that agree.

There needs to be a defined and separate limitations section within the

discussion, specifically there could be some discussion of how some of the

artefacts used could be leading – what was chosen to represent the ‘who is

fitter’ Q that was given etc. and/or the consideration of different ages, being at a

different life stage and how this may impact perceptions. What I mean is how

can you compare the change over time from 10 to 13 years old versus 12 to 17

years old… this is a different transition, could details/understanding/voices be

lost? The authors own reflections of the potential limitations and what the study

offers should therefore be considered and included.

Thank you for these comments. I revisited the data to look for potential differentiating factors and found that participants who reported barrier/facilitator changes were, on average, one year older than those who did not. This has now been added as a short paragraph in the results section. While this may suggest that changes in the experience of PA occur more frequently in late adolescence, the age ranges of changers and non-changers still overlap considerably (13–18 years for changers and 13–16 years for non-changers).

Of course this additional finding does not fully resolve this comment. Therefore, we have addressed both points, the potential influence of our study and stimuli design, and the difference between earlier and later adolescence, in the limitations section. There, we discuss how different stages of adolescence will have specific characteristics, acknowledge the influence of our own perspectives on the study design and interpretation, and suggest future directions, such as involving adolescents more actively as co-researchers.
